# Supplementary figures and images for: Human papillomavirus type 38 alters wild-type p53 activity to promote cell proliferation via the downregulation of integrin alpha 1 expression
Source: PLoS Pathog. 2020 Aug 19;16(8):e1008792. doi: 10.1371/journal.ppat.1008792 (PMC7458291; doi:10.1371/journal.ppat.1008792)

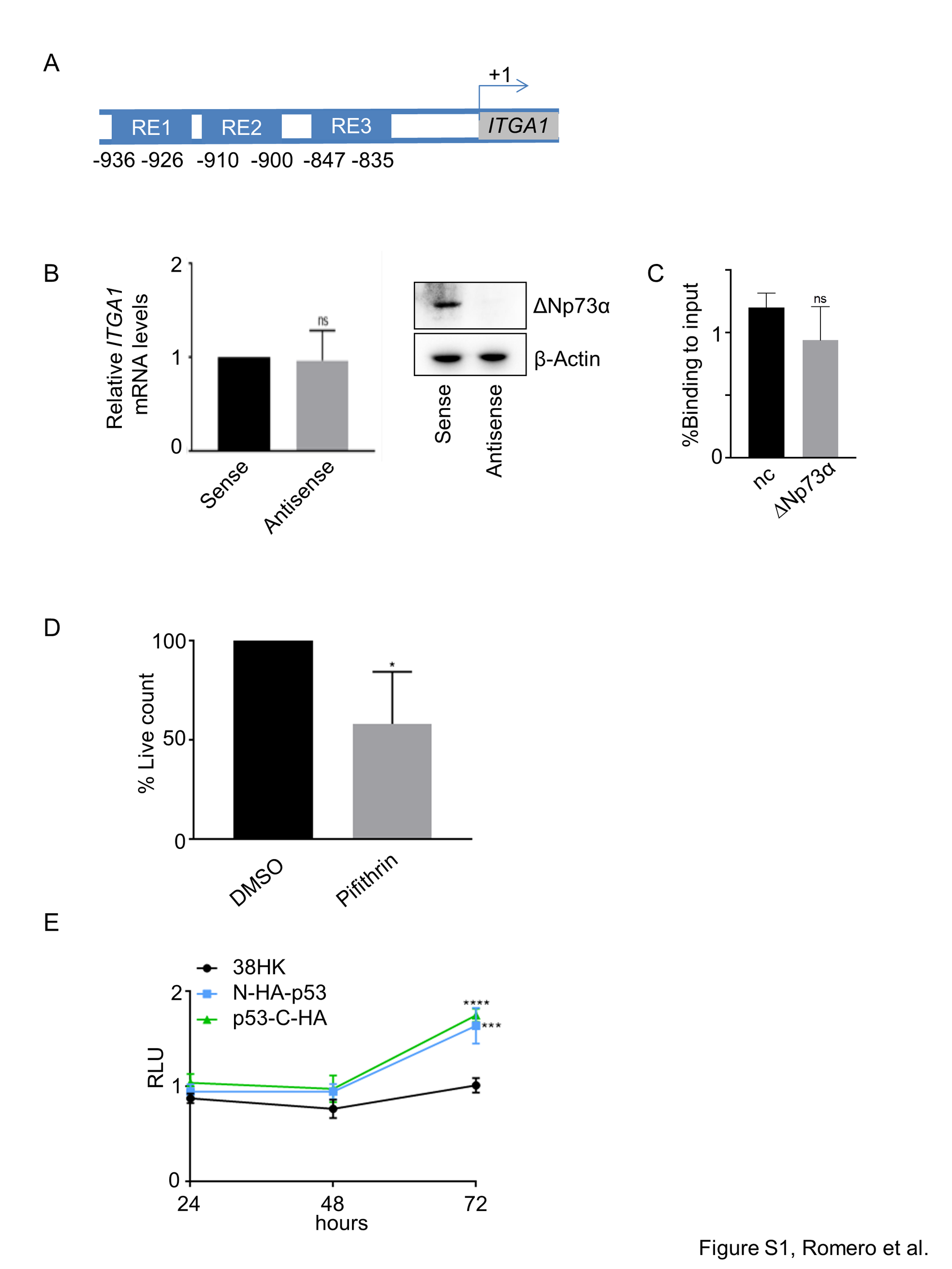

Supplement: S1 Fig — (A) Schematic representation of the ITGA1 promoter. p53 REs were predicted using TFBind and JASPAR software. (B) 38HK cells were transfected with sense (control) and antisense oligonucleotides against ΔNp73α. After 24 h, cells were collected and processed for RT-qPCR (left) or IB (right) (n = 3). ns, not significant. (C) Chromatin from 38HK was processed for ChIP experiments using p73 antibody. Results were obtained by qPCR with primers spanning p53RE2 or a negative control region (nc) (S2 Table). Error bars indicate standard deviations from 2 independent experiments performed in duplicate. ns, not significant. (D) 38HK cells were treated with cyclic pifithrin-α hydrobromide or DMSO as a control for 24 h, stained with trypan blue, and counted. The histograms represent the means of 4 independent experiments. *, p<0.05; **, p<0.01. (E) Cells expressing N-HA-p53 or p53-C-HA were seeded into 96-well plates. After 24, 48 and 72 h, cells were incubated with 20 μL of MTS solution for 2 h. Absorbance was obtained at 490 nm. Data shown are the means of 2 independent experiments performed in duplicate. ***, p<0.001; ****, p<0.0001. DOI: 10.6084/m9.figshare.12732737 (TIF) [file ppat.1008792.s001.tif]
