# Supplementary material for: Human papillomavirus type 38 alters wild-type p53 activity to promote cell proliferation via the downregulation of integrin alpha 1 expression
Source: PLoS Pathog. 2020 Aug 19;16(8):e1008792. doi: 10.1371/journal.ppat.1008792 (PMC7458291; doi:10.1371/journal.ppat.1008792)

Figure 1C

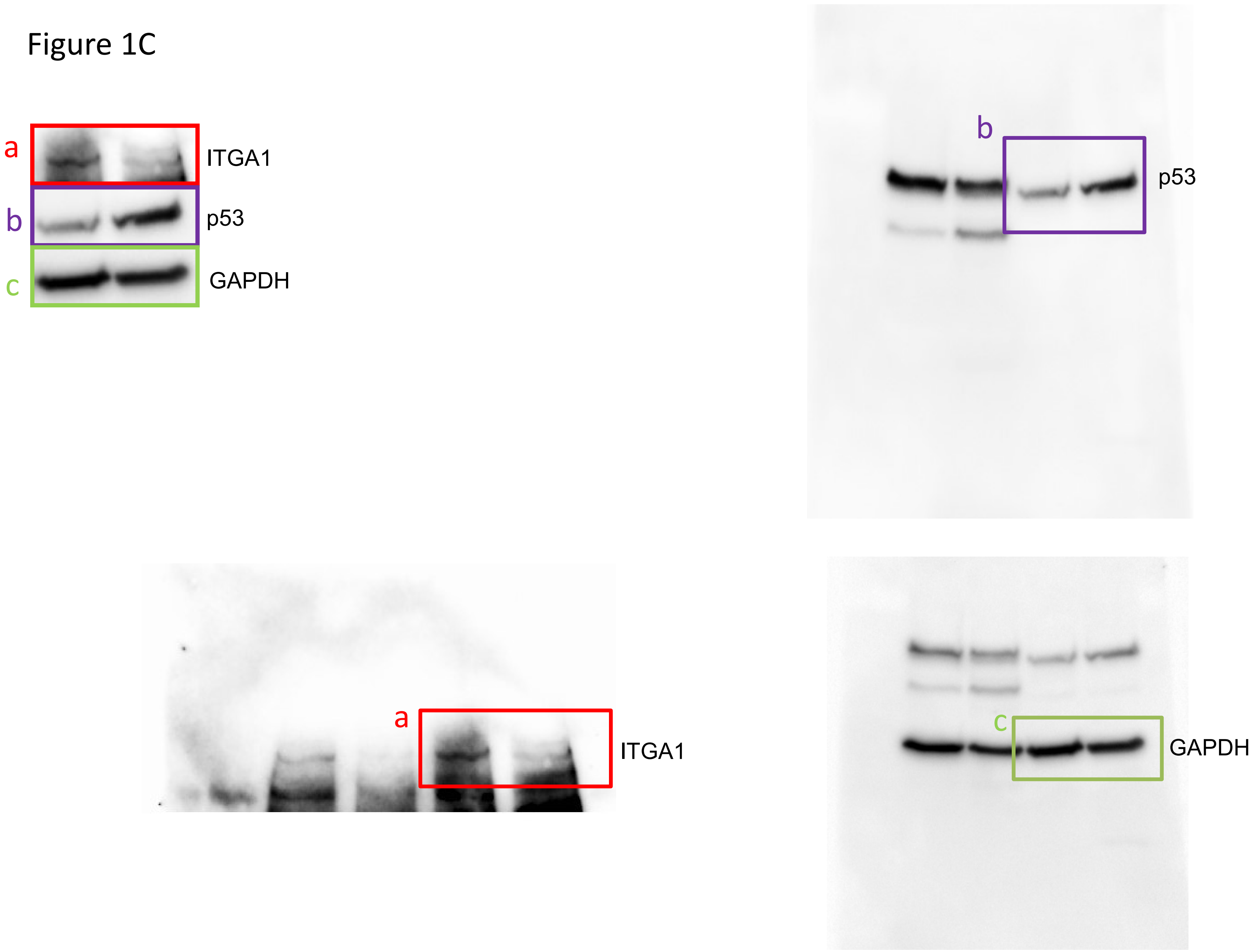

Figure 1E

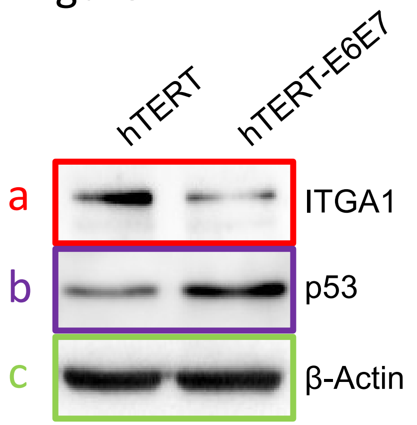

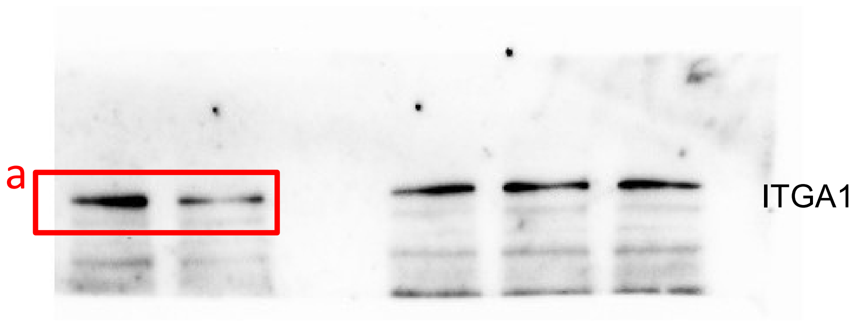

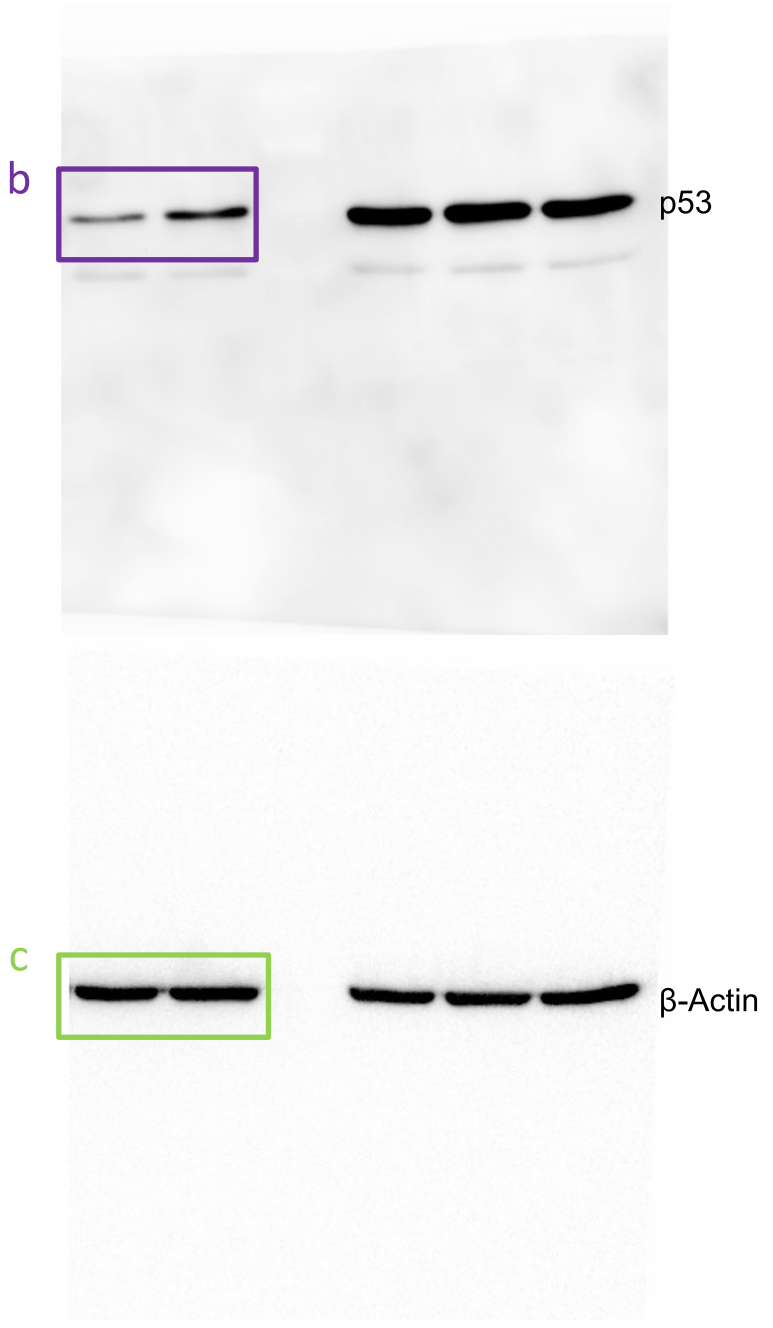

Figure 2A

B

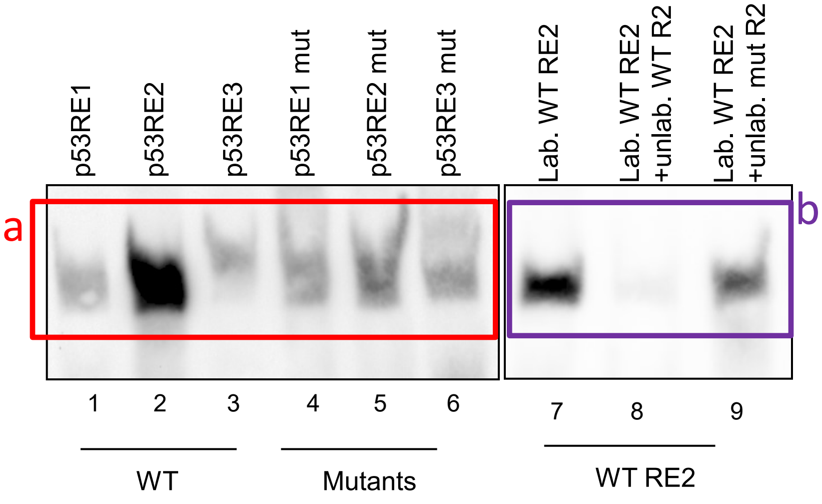

p53RE1
p53RE2
p53RE3
p53RE1 mut
p53RE2 mut
p53RE3 mut
Lab. WT RE2
Lab. WT RE2 +unlab. WT R2
Lab. WT RE2 +unlab. mut R2
a
b
1
2
3
4
5
6
7
8
9
WT
Mutants
WT RE2

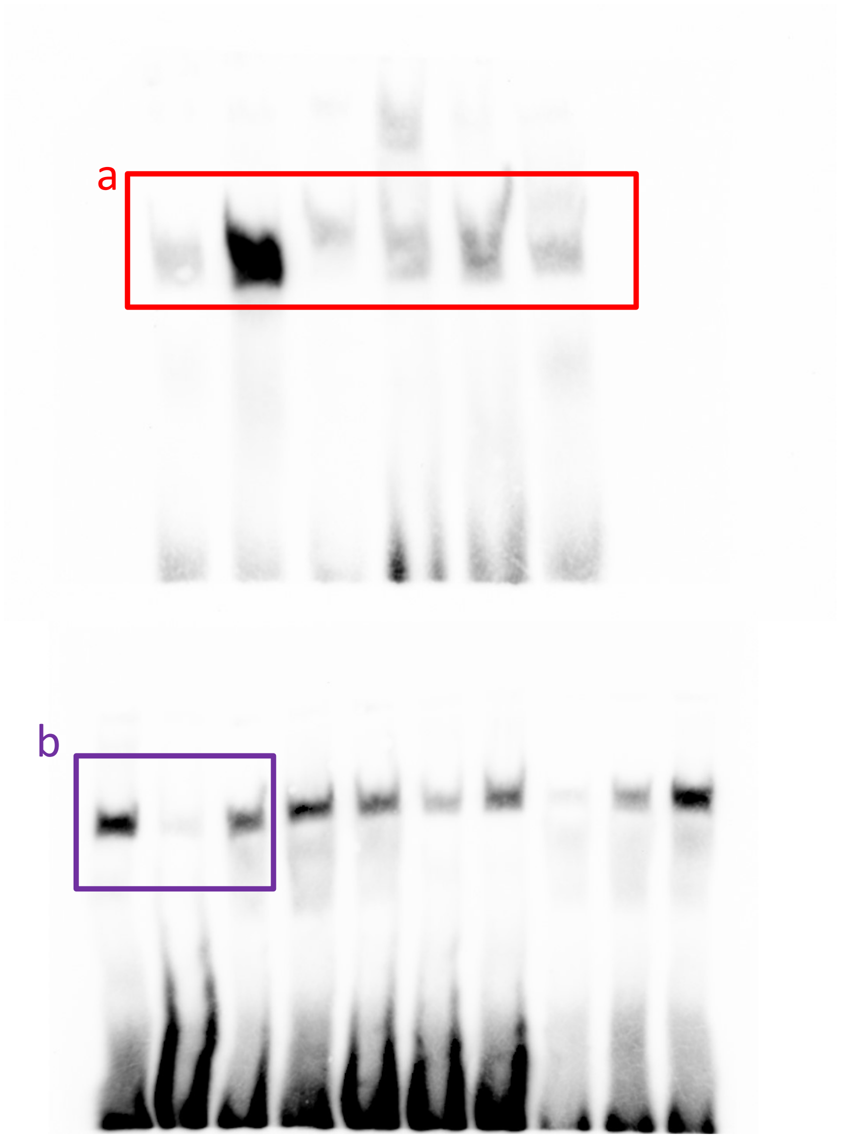

a
b

Figure 2D

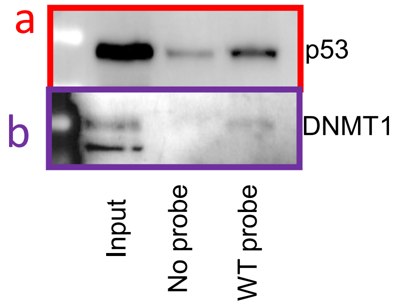

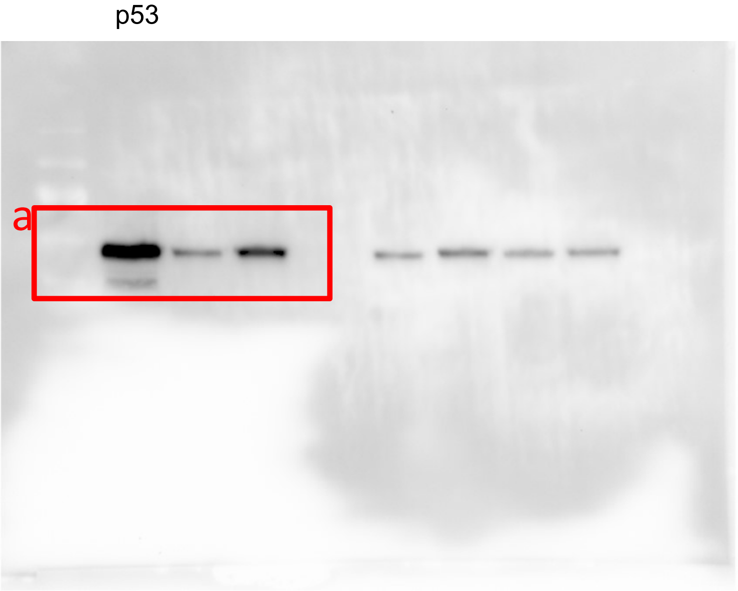

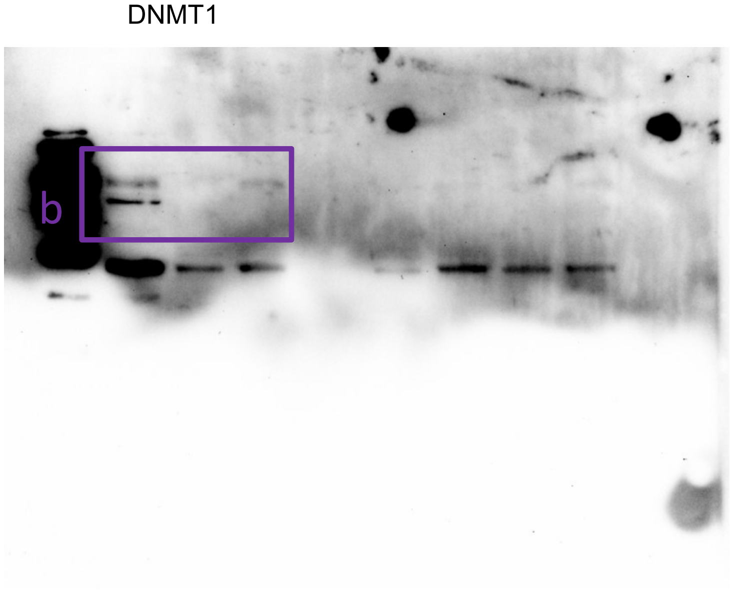

Figure 2H

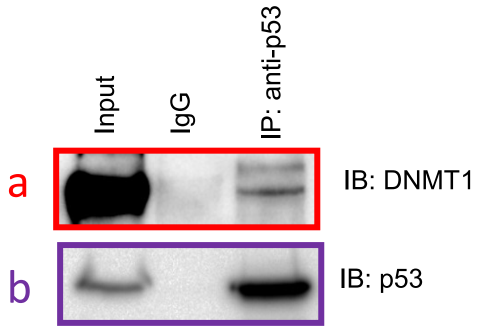

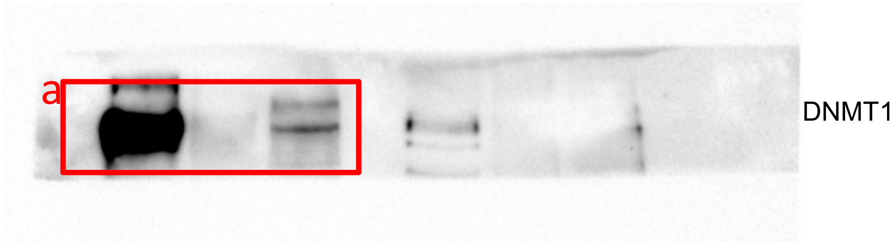

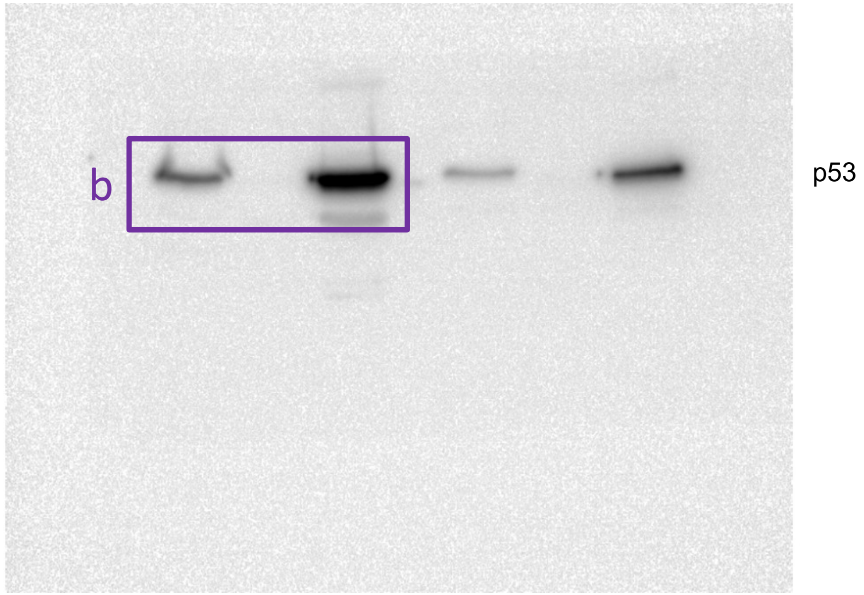

Figure 2I

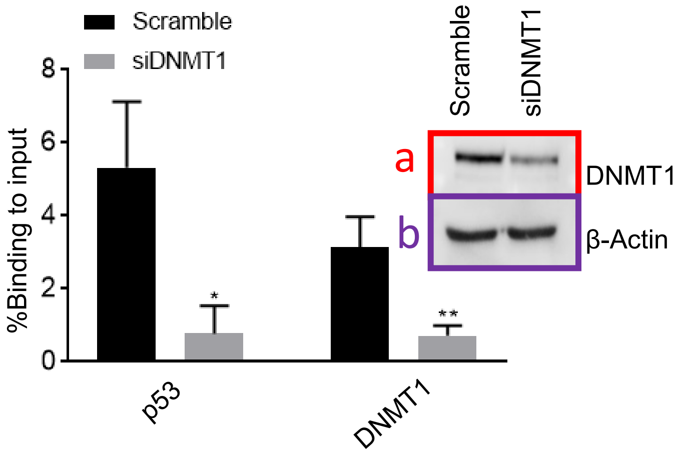

Scramble
siDNMT1
%Binding to input
8
6
4
2
0
*
**
p53
DNMT1
Scramble
siDNMT1
a
b
DNMT1
β-Actin

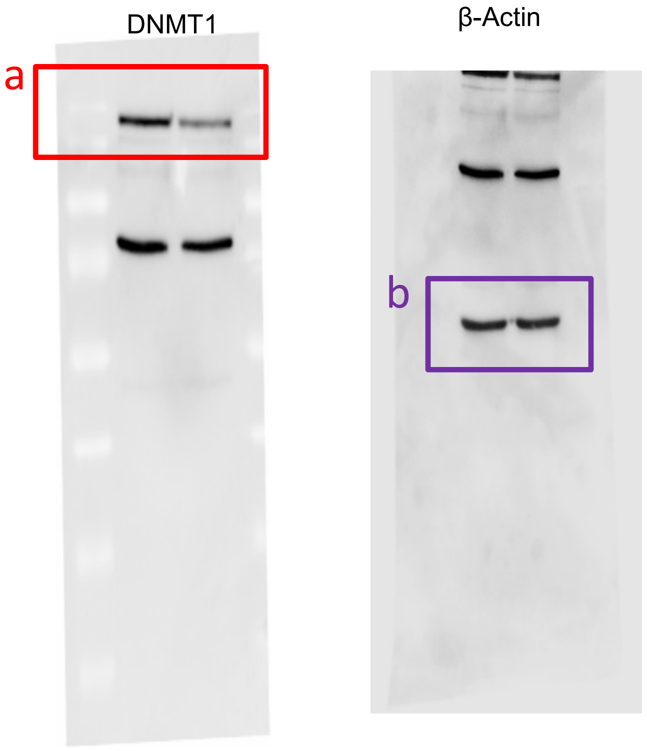

DNMT1
β-Actin
a
b

Figure 3C

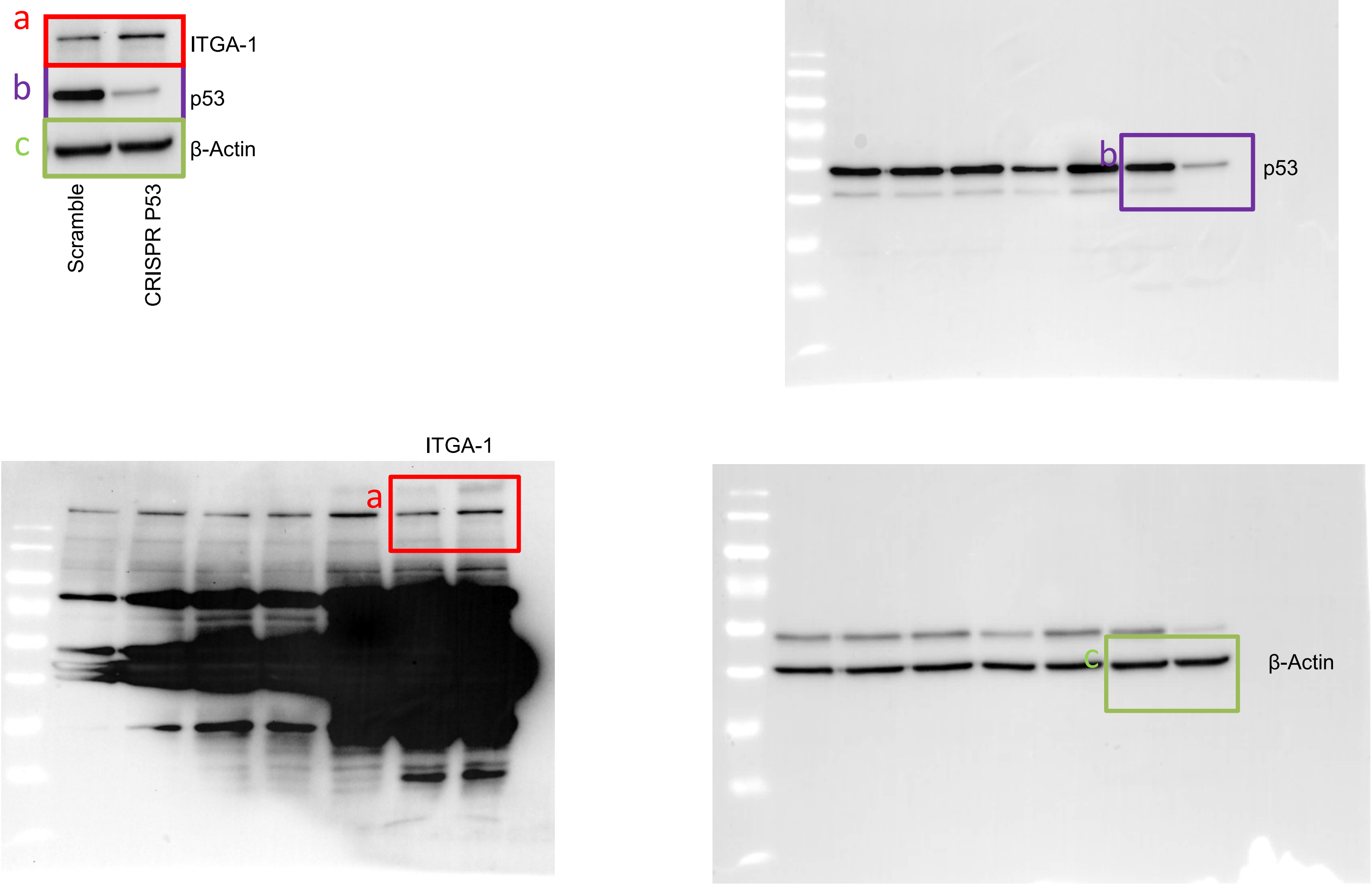
a
ITGA-1
b
p53
c
β-Actin
Scramble
CRISPR P53
b
p53
ITGA-1
a
c
β-Actin

Figure 3D

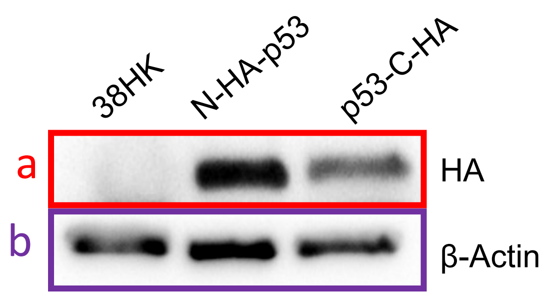

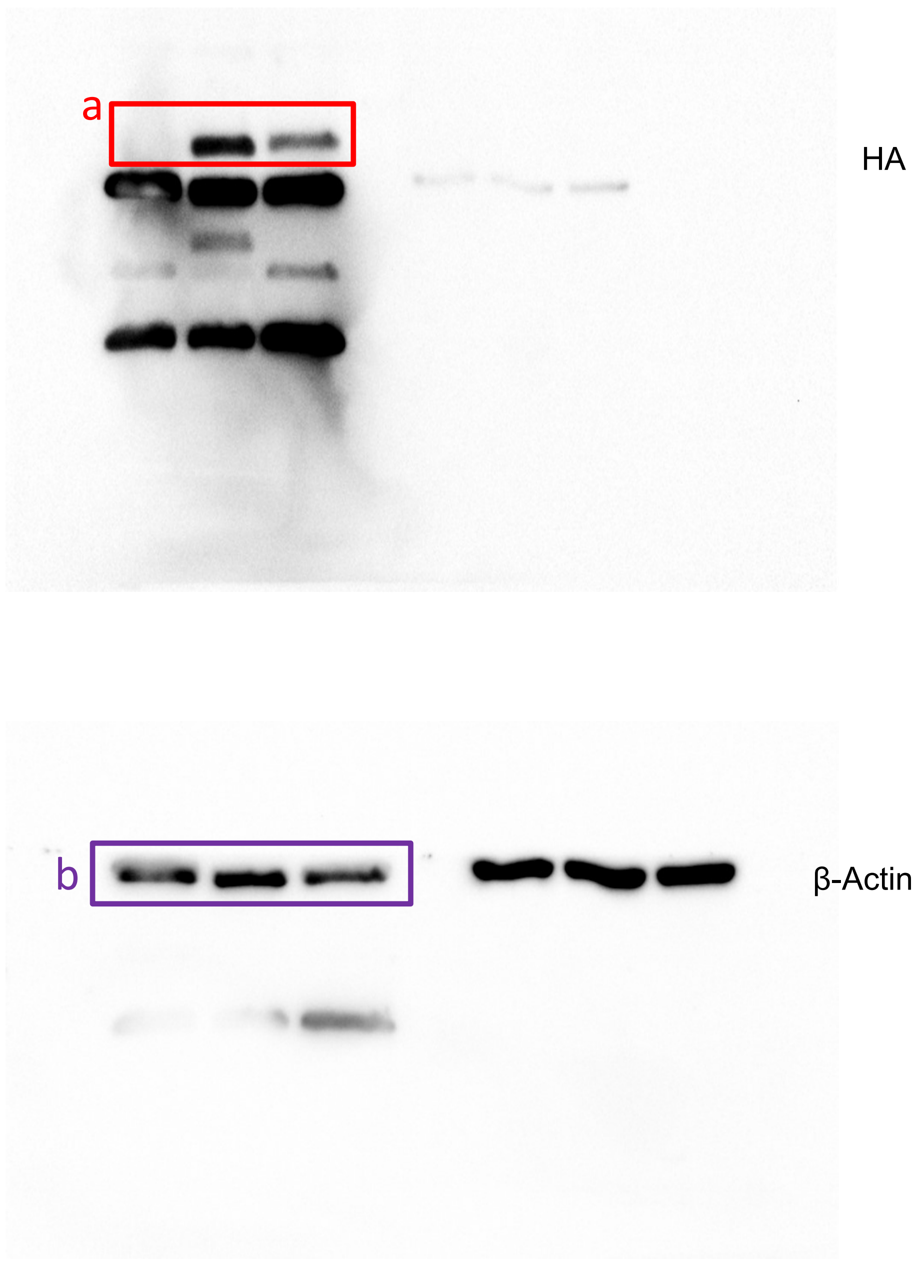

Figure 4A

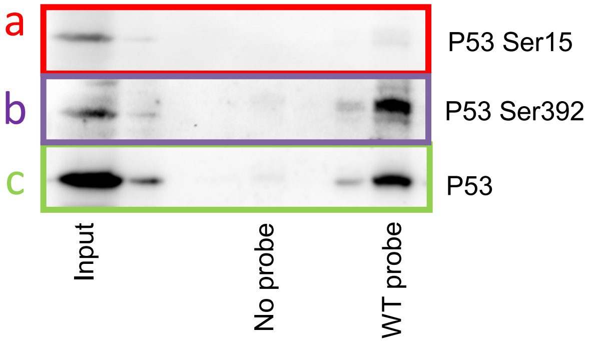
a
b
c
P53 Ser15
P53 Ser392
P53
Input
No probe
WT probe

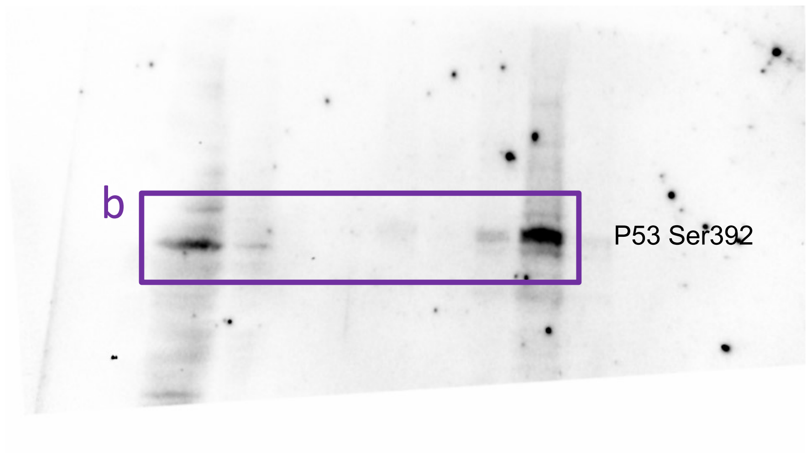
b
P53 Ser392

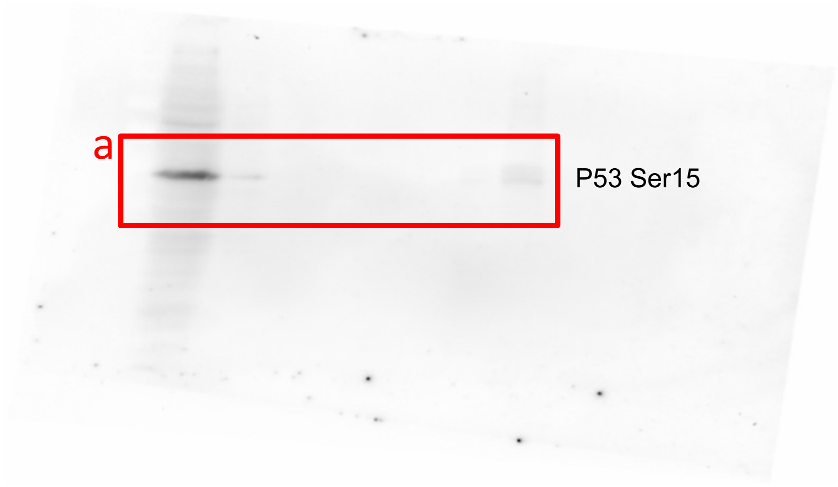
a
P53 Ser15

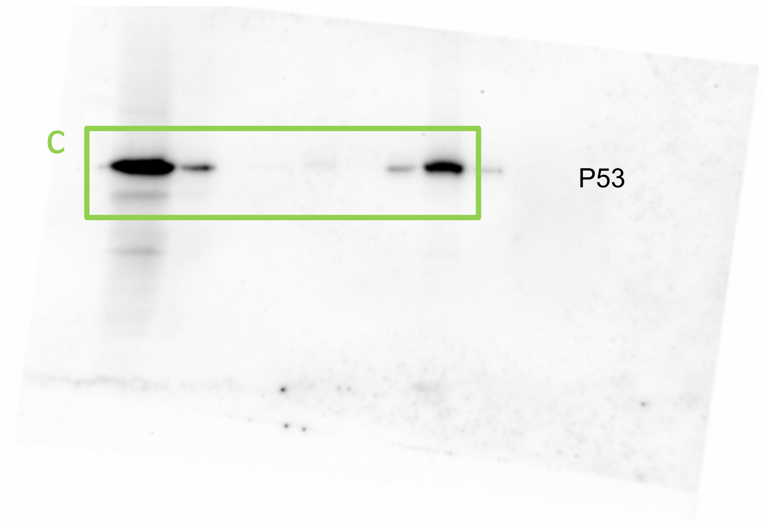
c
P53

Figure 4B

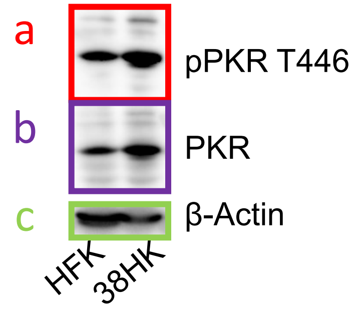

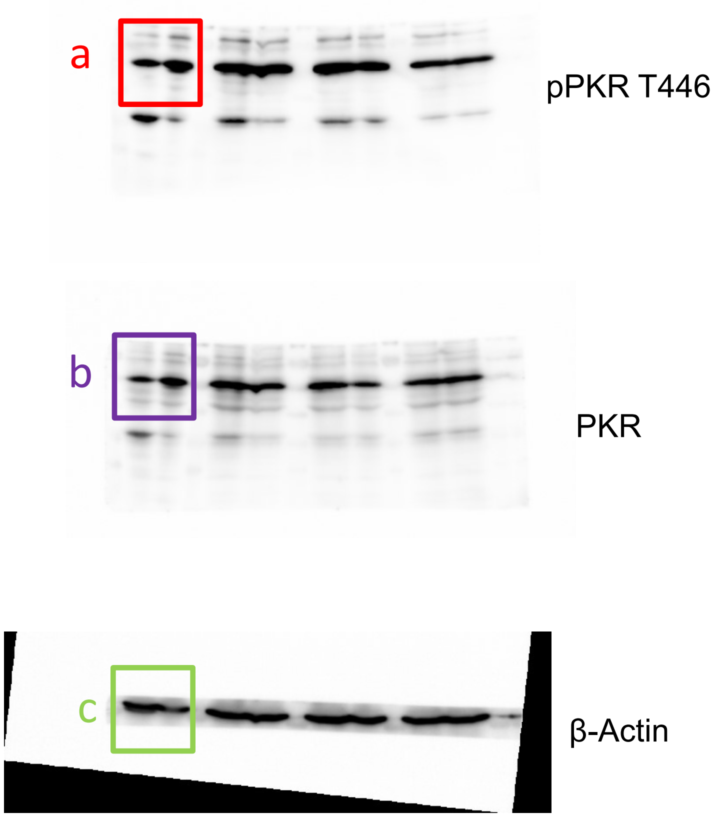

Figure 4C

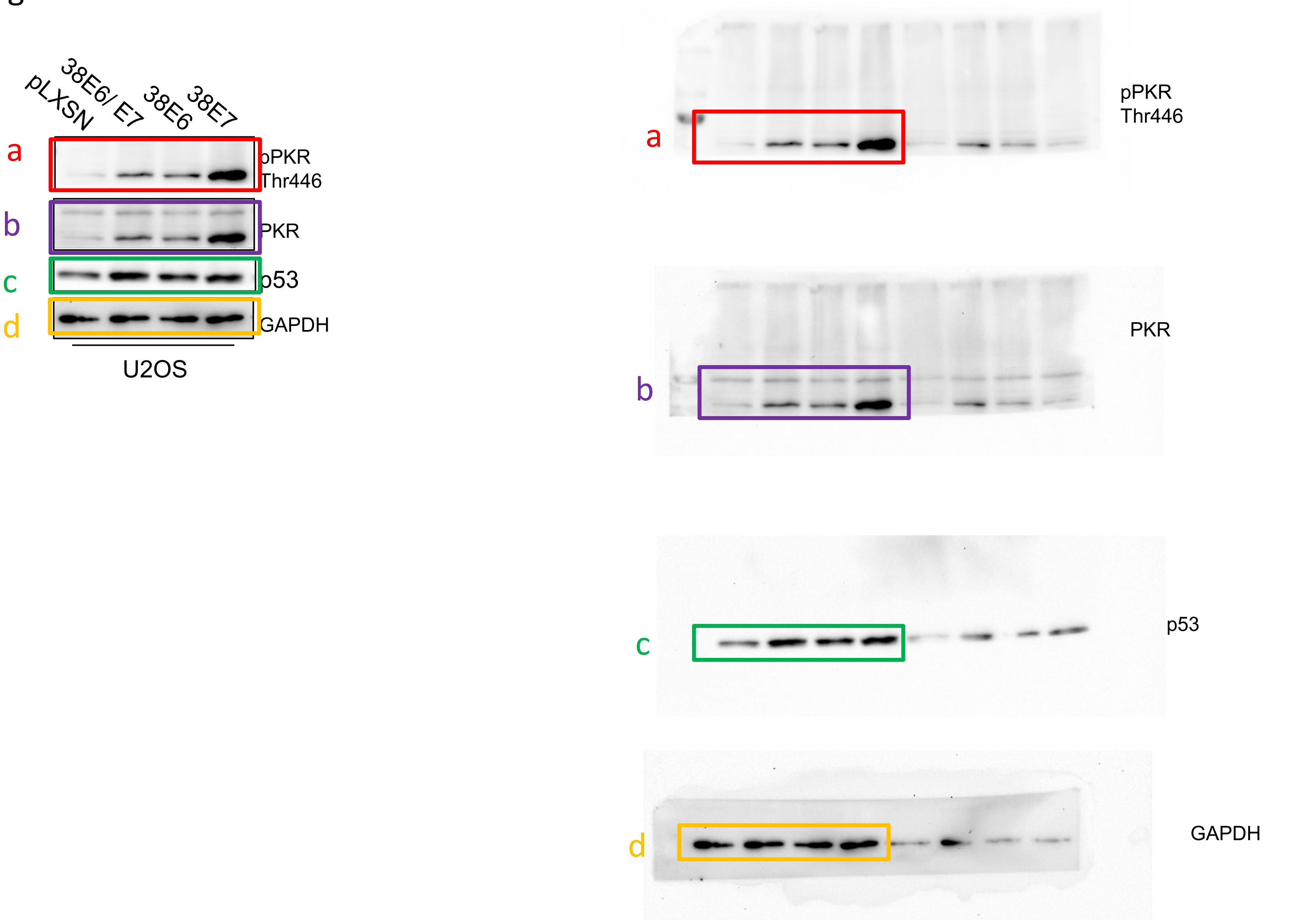
pLXSN
38E6/ E7
38E6
38E7
a
pPKR Thr446
b
PKR
c
p53
d
GAPDH
U2OS
a
pPKR Thr446
b
PKR
c
p53
d
GAPDH

Figure 4D

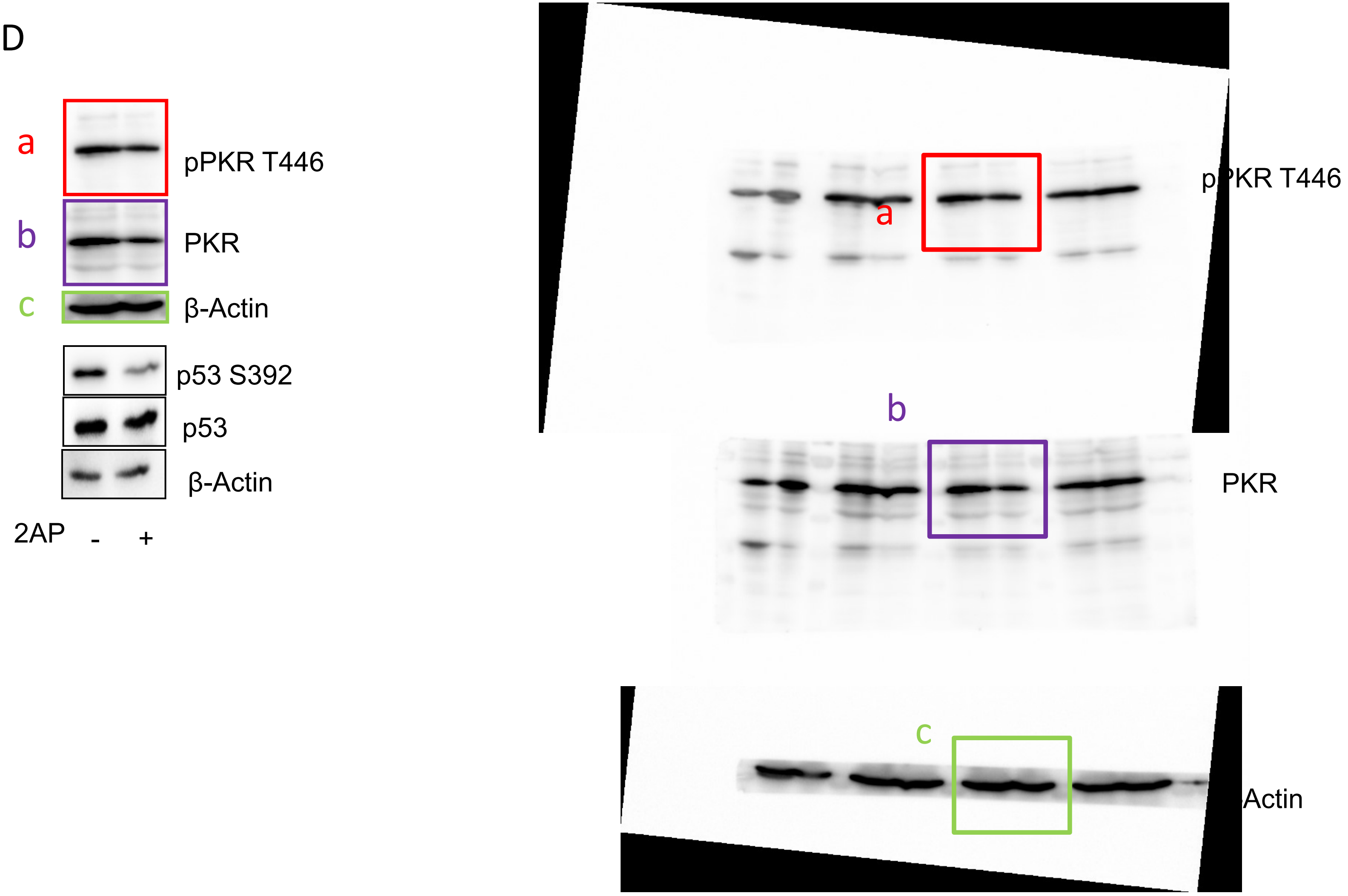

a
pPKR T446
b
PKR
c
β-Actin
p53 S392
p53
β-Actin
2AP
-
+
pPKR T446
PKR
Actin

Figure 4D BIS

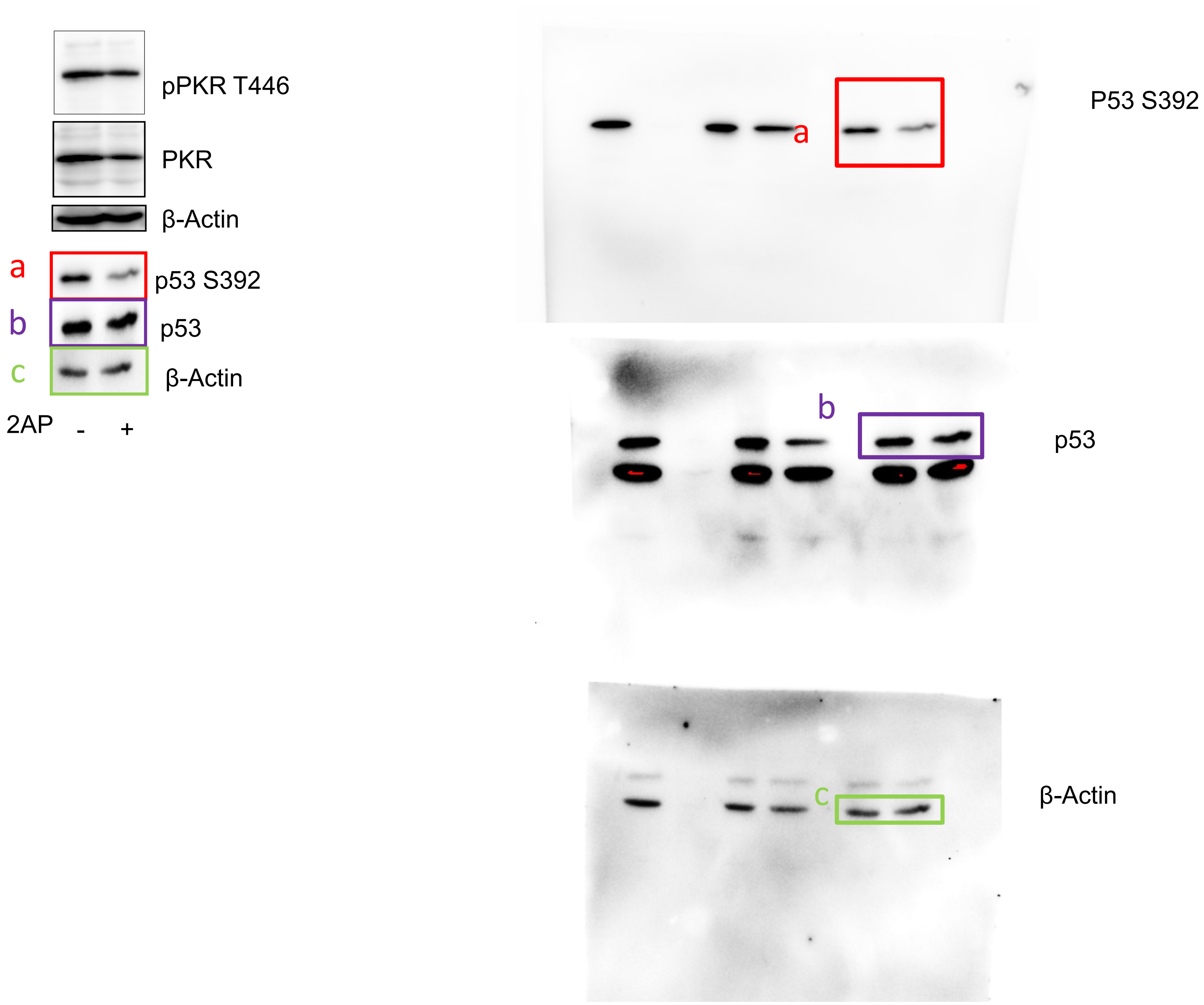

Figure 4E

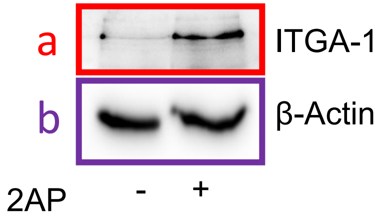

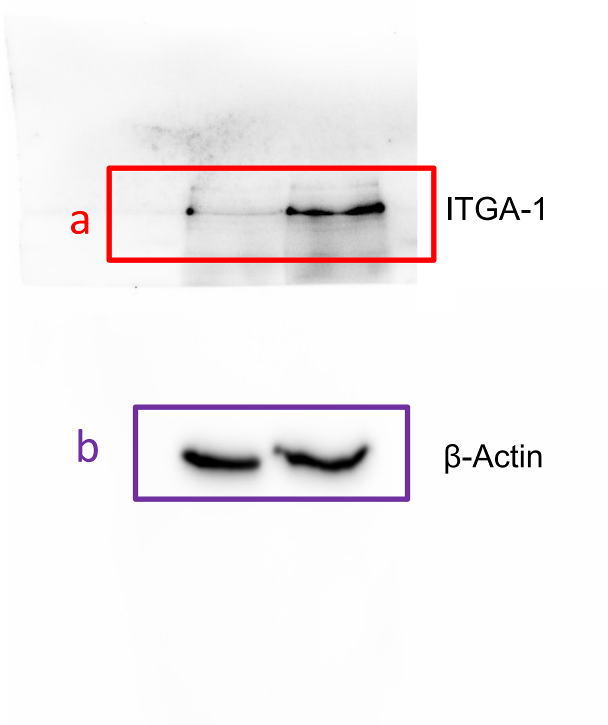

Figure 5A

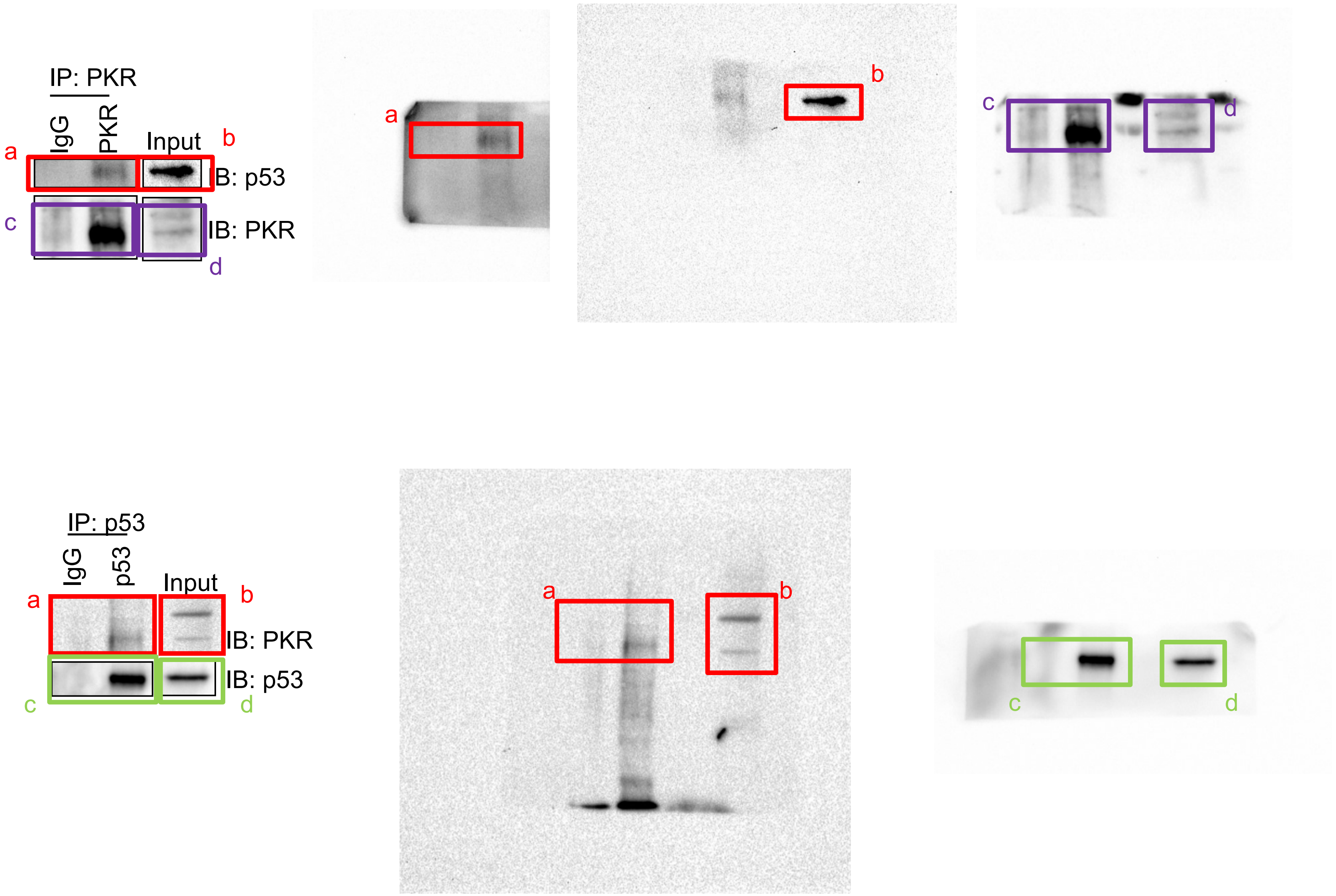

IP: PKR
IgG
PKR
Input
a
b
IB: p53
c
IB: PKR
d
IP: p53
IgG
p53
Input
IB: PKR
IB: p53

Figure 5B

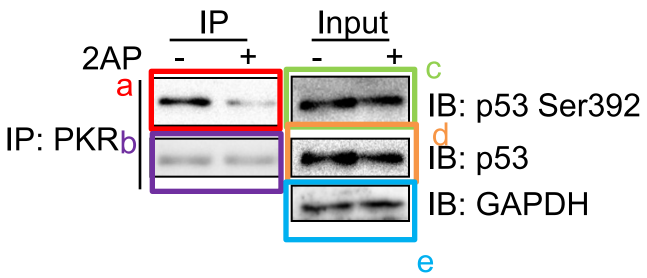

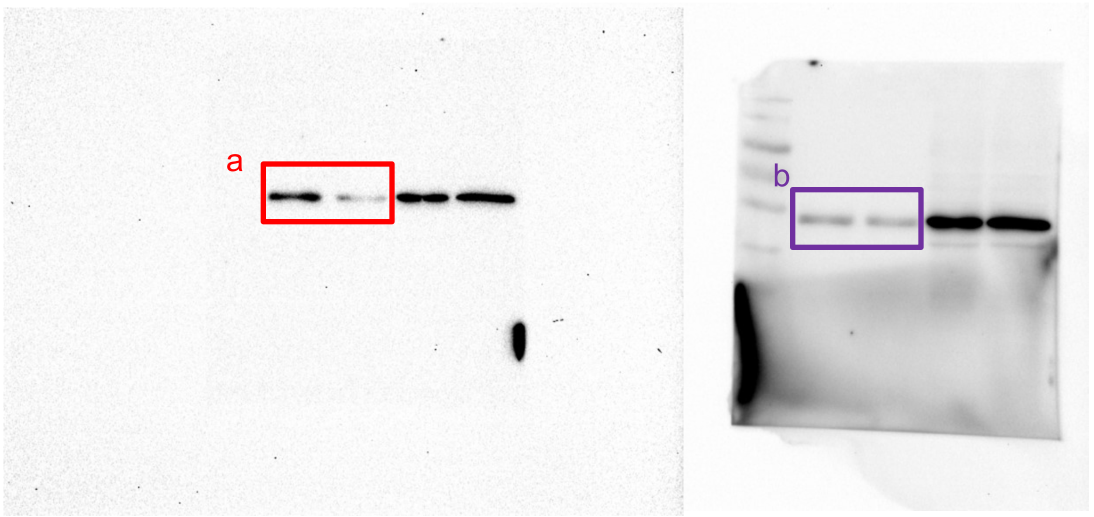

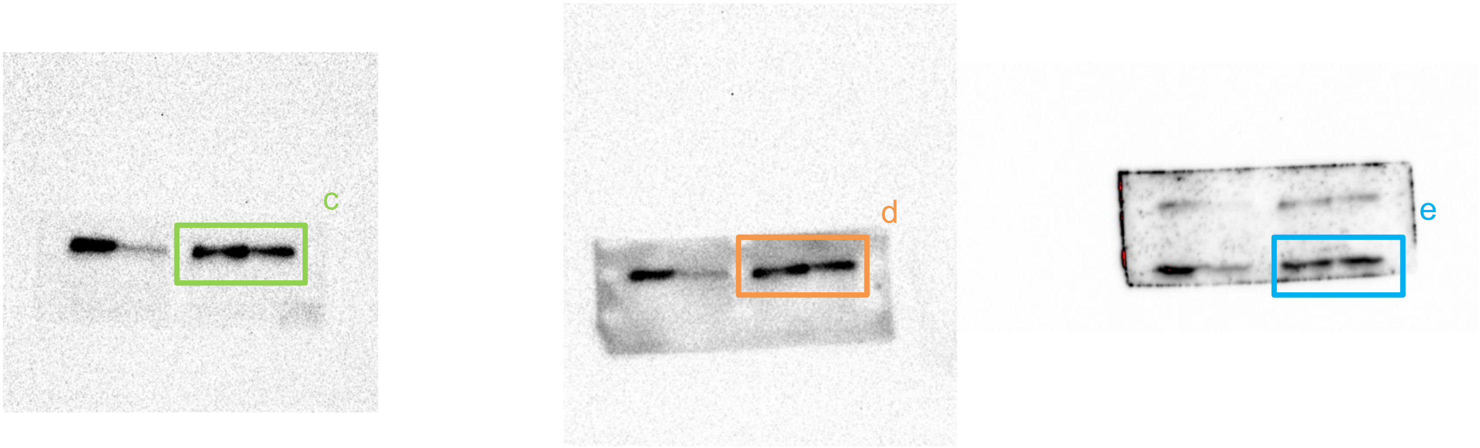

Figure 5D (left panel)

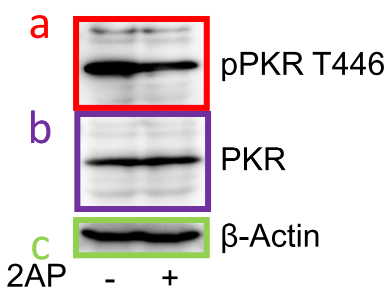

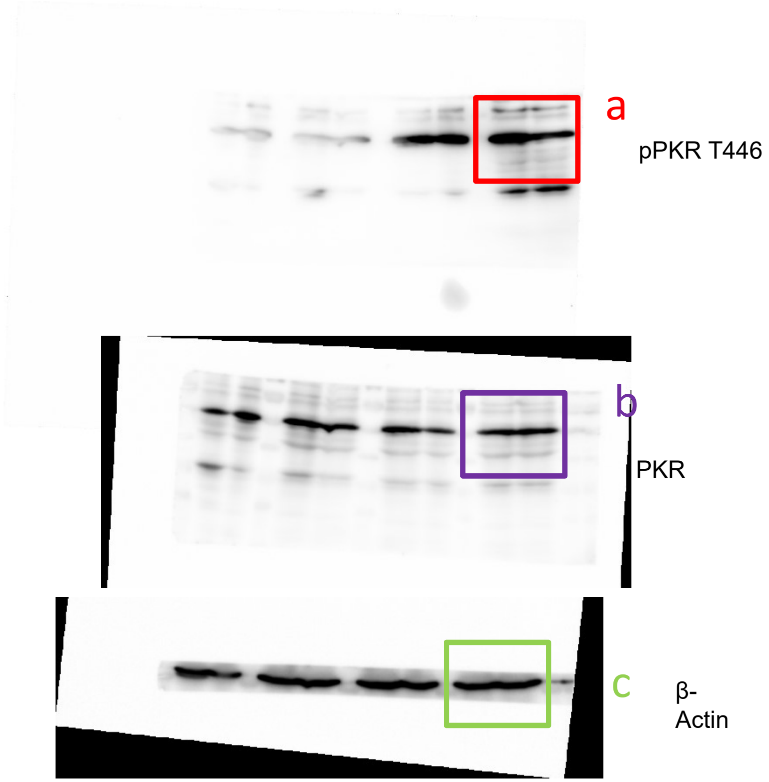

Figure 5D (right panel)

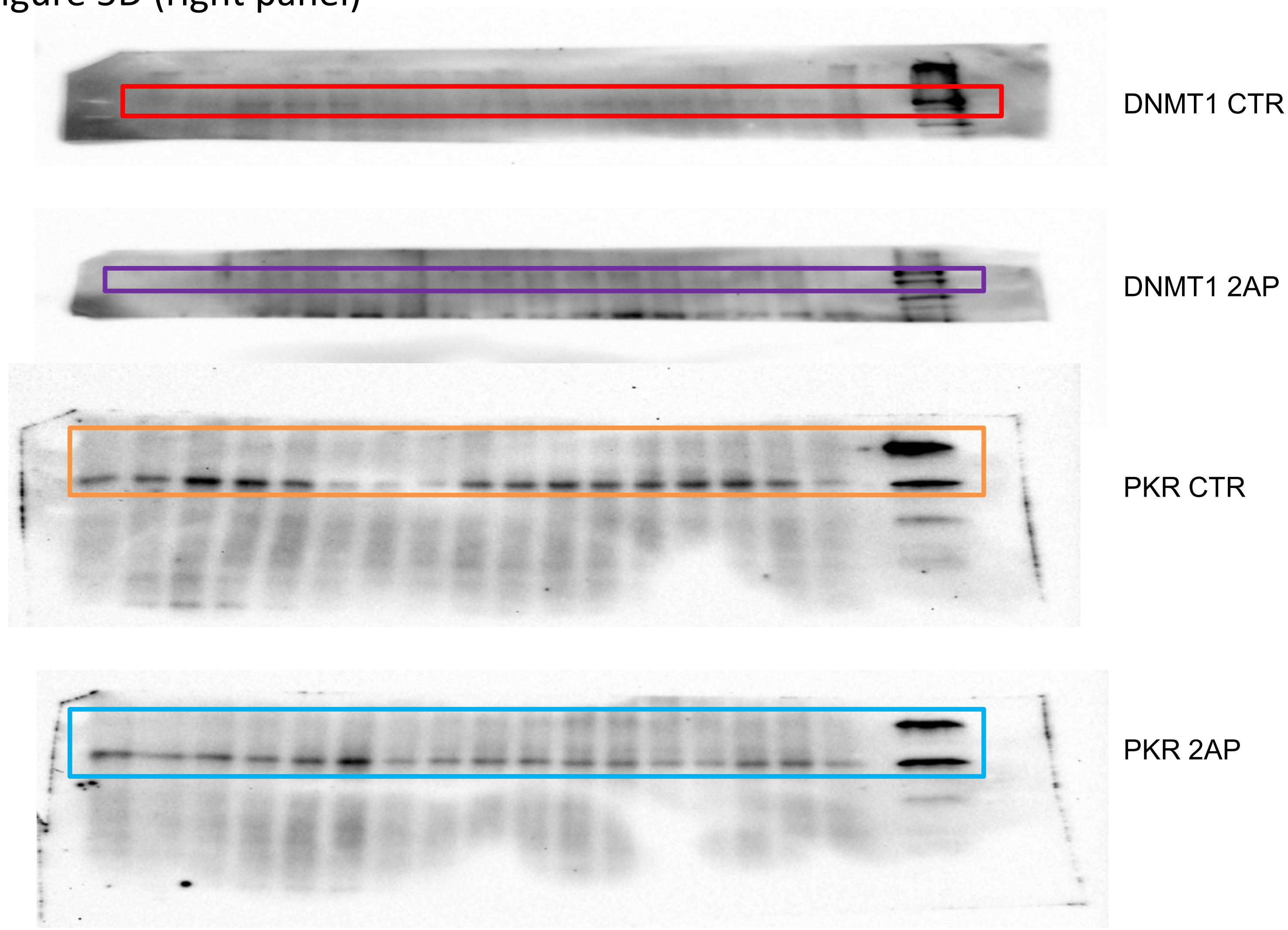

Figure 5D (right panel)

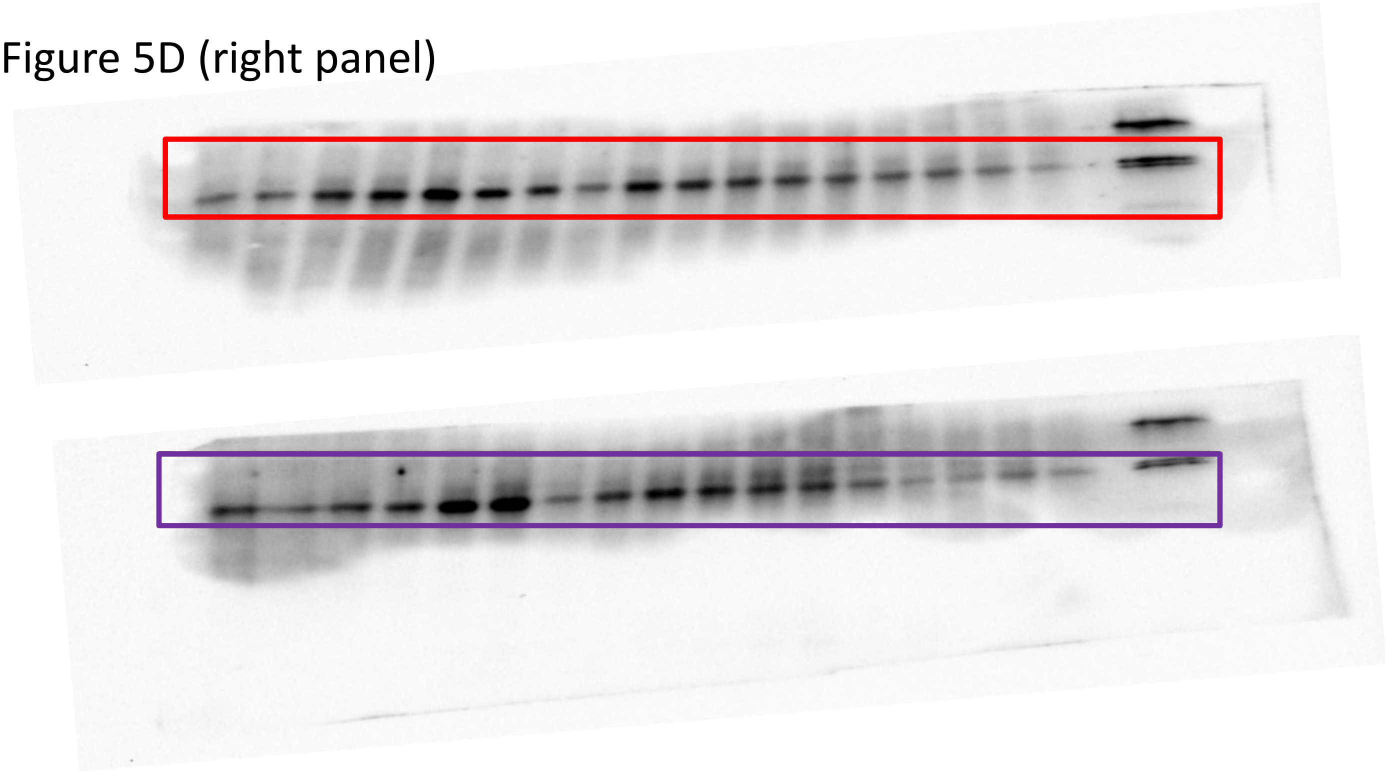

P53 CTR

P53 2AP

Figure 6C

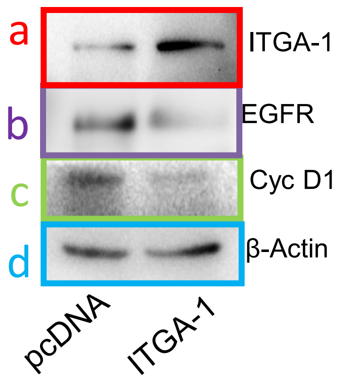

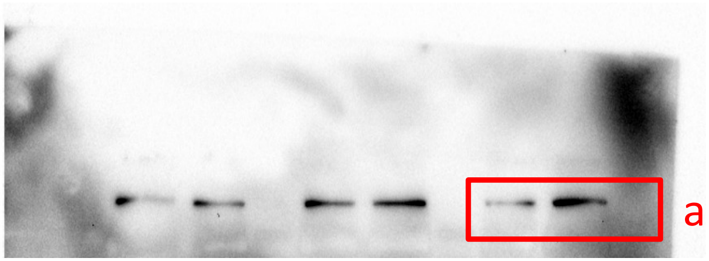

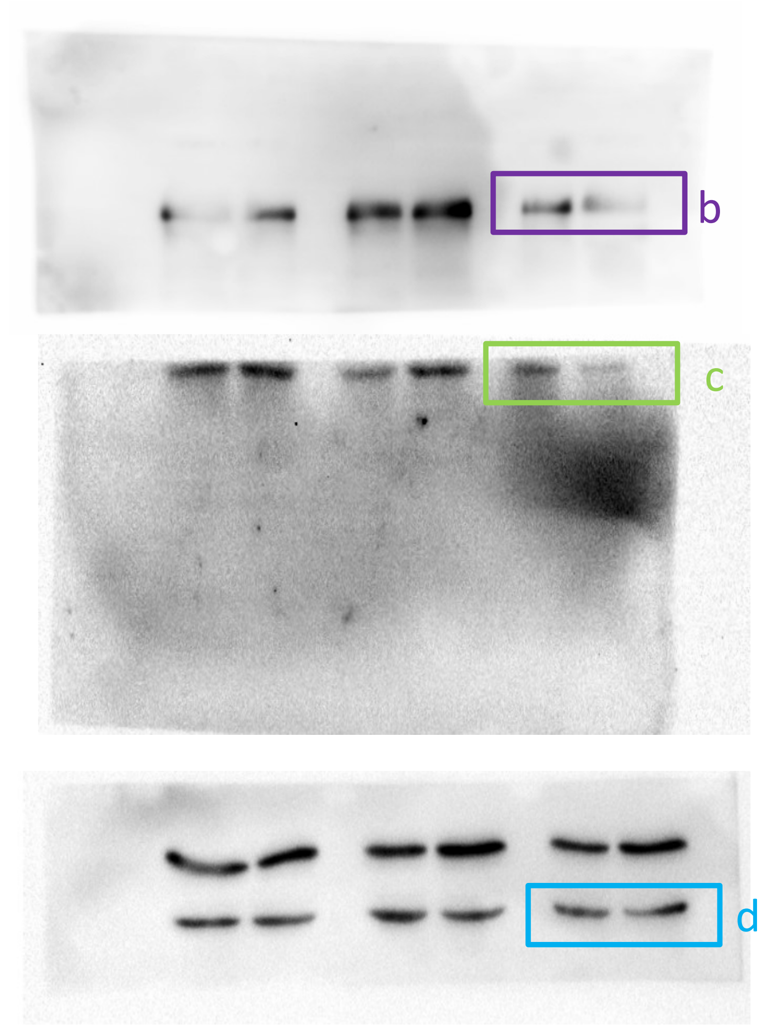

Figure 7 A

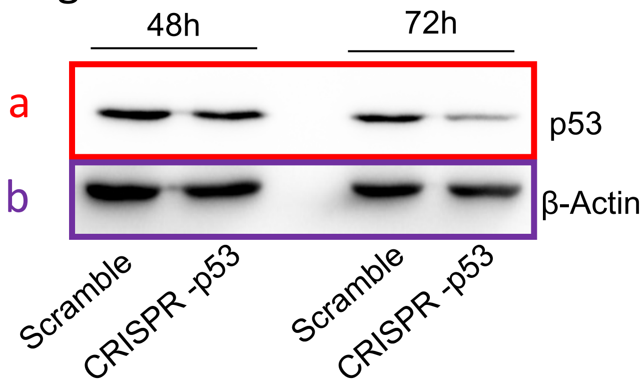
48h
72h
a
b
p53
β-Actin
Scramble
CRISPR -p53
Scramble
CRISPR -p53

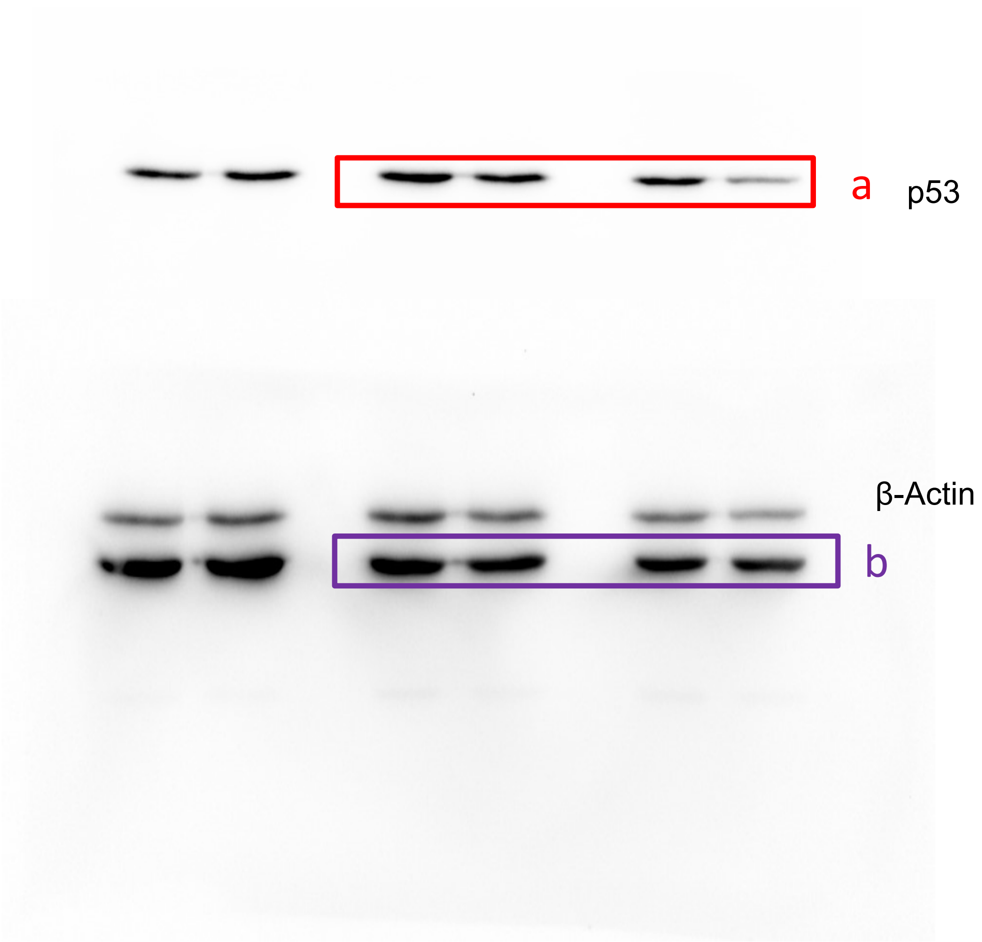
a
p53
β-Actin
b

Figure 7E

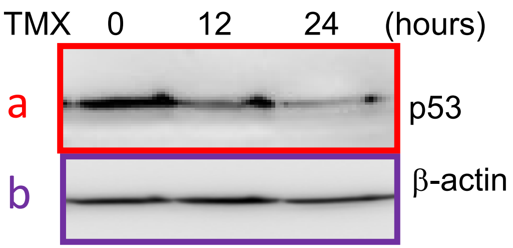

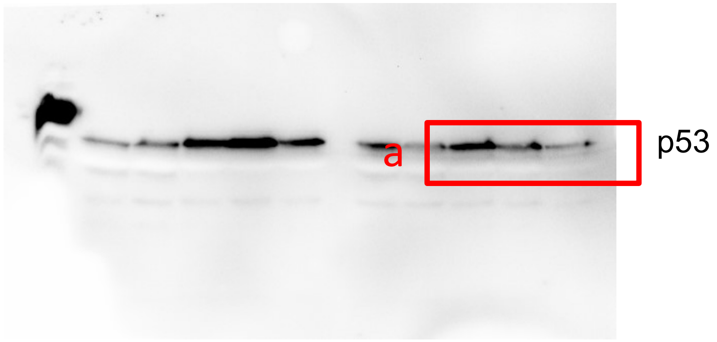

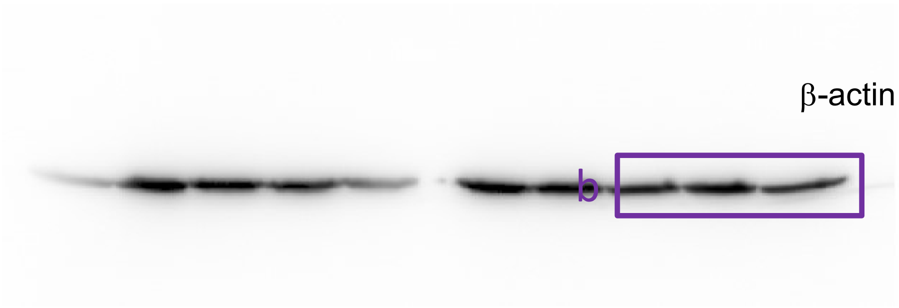

Figure 7F

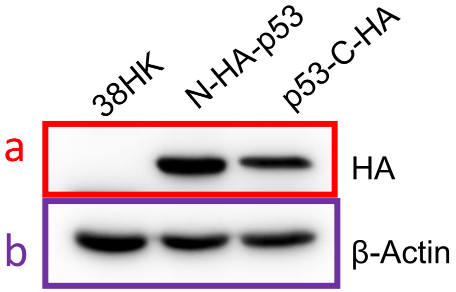

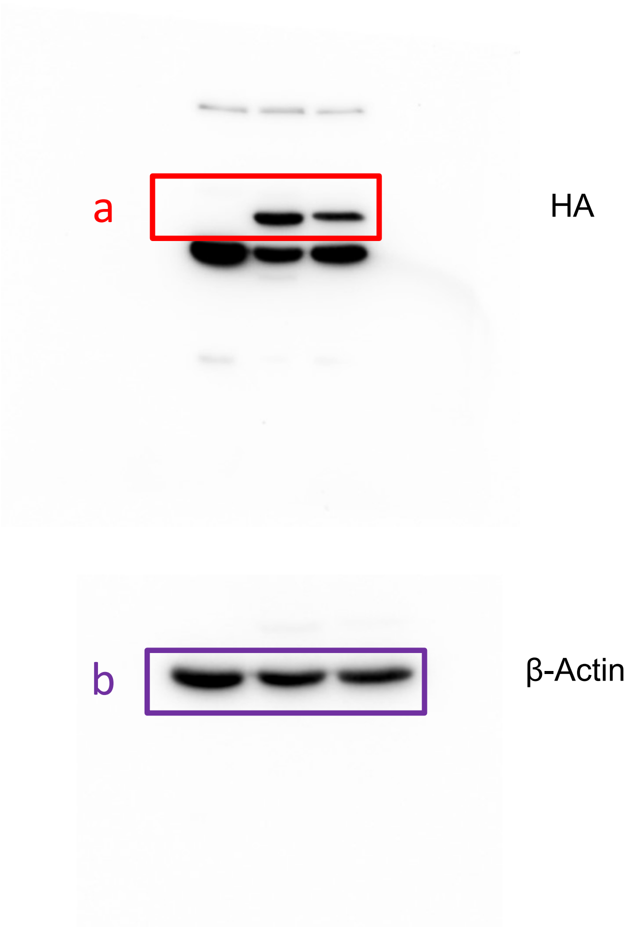

Figure S1B

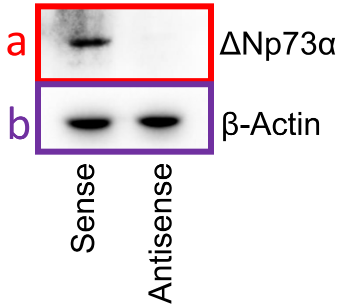

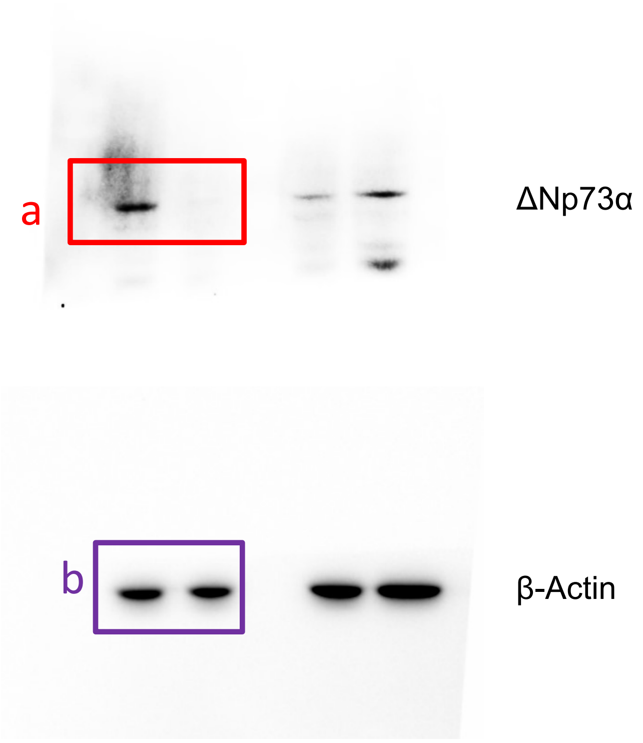

Supplement: S2 Fig — (PDF) [file ppat.1008792.s002.pdf]
