# Supplementary material for: Human papillomavirus type 38 alters wild-type p53 activity to promote cell proliferation via the downregulation of integrin alpha 1 expression
Source: PLoS Pathog. 2020 Aug 19;16(8):e1008792. doi: 10.1371/journal.ppat.1008792 (PMC7458291; doi:10.1371/journal.ppat.1008792)
Supplement: S1 Table — DOI: 10.6084/m9.figshare.12733442. (DOCX) [file ppat.1008792.s003.docx]

S1 Table. Sequences of siRNA and CRISPR/Cas9 vectors used for gene silencing

| **Target** | **siRNA sequence or description** |
| --- | --- |
| Scrambled RNA (negative control) | 5′-GGUGGAAGAGGUGGUGAGC-3′ |
| DNMT1 | 5′-UUGGAGAACGGUGCUCAUGCUUACA-3′  5′-GUAAGCAUGAGCACCGUUCUCCAA-3′ |
| p53 vector #1 | F: 5′-TCCATTGCTTGGGACGGCAAGTTTT-3′ |
|  | R: 5′-TTGCCGTCCCAAGCAATGGACGGTG-3′ |
| p53 vector #2 | F: 5′-CCATTGTTCAATATCGTCCGGTTTT-3′ |
|  | R: 5′-CGGACGATATTGAACAATGGCGGTG-3′ |
| p53 vector #3 | F: 5′-CTCGGATAAGATGCTGAGGAGTTTT-3′ |
|  | R: 5′-TCCTCAGCATCTTATCCGAGCGGTG-3′ |
| p53 vector #4 | F: 5′-CACTTTTCGACATAGTGTGGGTTTT-3′ |
|  | R: 5′-CCACACTATGTCGAAAAGTGCGGTG-3′ |
| Scrambled vector | F: 5′-GGATGGACGGTAGAGGTGGGTTTT-3′ |
|  | R: 5′-CCACCTCTACCGTCCATCCCGGTG-3′ |
