# Supplementary material for: Human papillomavirus type 38 alters wild-type p53 activity to promote cell proliferation via the downregulation of integrin alpha 1 expression
Source: PLoS Pathog. 2020 Aug 19;16(8):e1008792. doi: 10.1371/journal.ppat.1008792 (PMC7458291; doi:10.1371/journal.ppat.1008792)
Supplement: S2 Table — DOI: 10.6084/m9.figshare.12733445. (DOCX) [file ppat.1008792.s004.docx]

S2 Table. Primers for RT-qPCR, ChIP and EMSA experiments.

| **Target** | **RT-PCR, ChIP and EMSA primers sequence** |
| --- | --- |
| *ITGA1* | Fw 5’-ATCTCGGCAGCACAATTCAT-3’  Rv 5’-CTTACCATCCCCACCTGATG-3’ |
| *ITGA2* | Fw 5’-GGAACGGGACTTTCGCAT-3’  Rv 5’-GGTACTTCGGCTTTCTCATCA-3’ |
| *ITGA5* | Fw 5’-AGCCTCAGAAGGAGGAGGAC-3’  Rv 5’-TTAATGGGGTGATTGGTGGT-3’ |
| *ITGA6* | Fw 5’-TTTATCGGTCTCGGGAGTTG-3’  Rv 5’-GGCCACTGAATGTTCAAGGT-3’ |
| *ITGB1* | Fw 5’-ATCTGCGAGTGTGGTGTCTG-3’  Rv 5’-GGGGTAATTTGTCCCGACTT-3’ |
| *ITGB8* | Fw 5’-AATTTGGTAGTGGAAGCCTATC-3’  Rv 5’- GTCACGTTTCTGCATCCTTC-3’ |
| *GAPDH* | Fw 5’- AAGGTGGTGAAGCAGGCGT-3’  Rv 5’- GAGGAGTGGGTGTCGCTGTT-3’ |
| *TP53* | Fw 5’-GATGAAGCTCCCAGAATGCC-3’  Rv 5’-CAAGAAGCCCAGACGGAAAC-3’ |
| *DNMT1* | Fw 5’-GAGGAAGCTGCTAAGGACTAGTTC-3’  Rv 5’-ACTCCACAATTTGATCACTAAATC-3’ |
| *CCND1* | Fw 5’-TGACCCCGCACGATTTCATT-3’  Rv 5’- CATGGAGGGCGGATTGGAAA-3’ |
| *EGFR* | Fw 5’-ACTTCAAAAACTGCACCTCCAT-3’  Rv 5’-AATCAGCAAAACCCTGTGATT-3’ |
| *Itga1 mouse* | Fw 5′-TGACGCTCCTTAAACTTCAAC-3′  Rv 5’-CCTCGTCTGATTCACAGCGT-3’ |
| *Gapdh mouse* | Fw 5’-GTGACCCCATGAGACACCTC-3’  Rv 5’- GTATGTCCAGGTGGCCGAC-3’ |
| ITGA1 RE1 | Fw 5’-TGACGCTCCTTAAACTTCAAC-3’  Rv 5’-ACGAGGGACAGGAAGGAAG-3’ |
| ITGA1 RE2 | Fw 5′-CTTCCTGTCCCTCGTGTTAC-3’  Rv 5′-CAAGAGTGCCACCCAAAGGA-3’ |
| ITGA1 RE3 | Fw 5’-TGTGCTACCTGTGTTGCGT-3’  Rv 5’-AGGAATCTGAGGGAAACAGT-3′ |
| Chr22 (negative control) | Fw 5’- GGTGCTCCTGGAAGCTGGGC -3’  Rv 5’- AAGGCAGCTGGCGTGAGGC-3’ |
| Chr6 (negative control) | Fw 5’-CCGGAAGCACTTCTCCTAGA-3’  Rv 5’-AAGAGAGAGCGGAAGTGACG-3’ |
| EMSA Btn-p53RE1 | Fw 5’-AACCCTGTGGGCTTCTTCCTTCCTG-3’  Rv 5’-CAGGAAGGAAGAAGCCCACAGGGTT-3’ |
| EMSA Btn-p53RE1 mut | Fw 5’-AACCCTGTGGTATTAATCCTTCCTG-3’  Rv 5’-CAGGAAGGATTAATACCACAGGGTT-3’ |
| EMSA Btn-p53RE2 | Fw 5’-CCTCGTGTTACCTGTTCTGTGCTAC-3’  Rv 5’-GTAGCACAGAACAGGTAACACGAGG-3’ |
| EMSA Btn-p53RE2 mut | Fw 5’-CCTCGTGTTTTCTAATCTGTGCTAC-3’  Rv 5’-GTAGCACAGATTAGAAAACACGAGG-3’ |
| EMSA Btn-p53RE3 | Fw 5’-TCTTGTCCTGCCTAGCCCTAG-3’  Rv 5’-CTAGGGCTAGGCAGGACAAGA-3’ |
| EMSA Btn-p53RE3 mut | Fw 5’-TCTTGTAATGCTAATCCCTAG-3’  Rv 5’-CTAGGGATTAGCATTACAAGA-3’ |
| DNA pulldown | Fw 5’-Btn ACCCTGTGGGCTTCTTCCT-3’  Rv 5’- AGGAATCTGAGGGAAACA-3’ |
