# Supplementary material for: Human papillomavirus type 38 alters wild-type p53 activity to promote cell proliferation via the downregulation of integrin alpha 1 expression
Source: PLoS Pathog. 2020 Aug 19;16(8):e1008792. doi: 10.1371/journal.ppat.1008792 (PMC7458291; doi:10.1371/journal.ppat.1008792)
Supplement: Attachment — Submitted filename: Rebuttal letter.doc [file ppat.1008792.s005.doc]

| 150 cours Albert Thomas 69372 Lyon cedex 08, France  Infections and Cancer Biology Group  Tel.: +33 4 72 73 81 90  Fax: +33 4 72 73 84 42  E-mail: icb@iarc.fr  http://www.iarc.fr |  |  |
| --- | --- | --- |
|  | |  |
| Ref.: re-submission modified version manuscript PPATHOGENS-D-20-00424 | | 15/04/2020 |

Dear Editor,

Many thanks for the excellent comments of the three reviewers. We were able to answer to the majority of the comments, also providing additional experiments as described below. Unfortunately, we are not currently able to perform the experiments with S392 p53 mutants (reviewer 2, comments 3, 6 and 7), since our Institute is partially operative due the Covid-19 situation.

Our responses

Reviewer #1:

Minor comments

**Comment 1: What is known about the effects of IGTA1 in high-risk HPV 16 or 18 positive cells? E6 reduces p53 levels in these cells so IGTA1 should go up yet cells do not exhibit reduced colony formation. This is puzzling and needs to be discussed.**

Our answer: We are not aware of studies that show a direct link between mucosal high-risk HPV types and ITGA1 expression. A recent study analyzed the difference in cellular gene expression in HPV-positive and HPV-negative oro-pharyngeal cancers (OPCs) using the Cancer Genome Atlas data (Gleber-, et al. JCI Insight. 2019 Jan 10;4(1):e124762). The findings showed that the expression of several integrins, including ITGA1, is deregulated in these cancers, being higher in HPV- positive OPCs than HPV- negative OPCs. These results suggest that the HPV38 ability to repress the ITGA1 expression is not shared with mucosal high-risk HPV type. Following the recommendation of the reviewer, these findings are discussed in the new version of the manuscript (page 15, lines 326-328)

**Comment 2: What role could IGTB1 which is also increased by HPV38 proteins?**

Our answer: The increase of ITGB1 showed in Fig. 1A is not significant. However, it is plausible to predict that the decrease of ITGA1 levels will impact on the biological functions of ITGB1, since the two proteins exert their activities by forming a dimer.

**Comment 3: How does overexpression of IGTA1 compare to the levels seen in normal keratinocytes? What are the increases in p53 in HPV 38 cells compared to normal keratinocytes?**

Our answer: Regarding the first question, we never compared in the same immunoblotting the ITGA1 protein levels in primary keratinocytes and HPV38 keratinocytes overexpressing ITGA1. However, since ITGA1 overexpression is regulated by CMV promoter, it plausible to hypothesize that the ectopic ITGA levels are higher than the natural levels.

Regarding p53 levels, we have now added the new panels in Figure 1 that show the increase of p53 levels induced by HPV38 E6/E7 in primary keratinocytes (Fig. 1C) and hTERT keratinocytes (Fig. 1E).

**Comment 4: How does the observation with the transgenic mice with the third cSCCC lesion that had increased ICTA1 and non-deleterious EGFR mutations fit in with their model for effects of IGTA1? Is p53 mutated in these cells?**

Our answer: Whole exome sequencing revealed that the third cSCC harbored wild-type Itga1 gene and a Trp53 mutated gene. Therefore, the increase of ITGA1 observed by in the third cSCC can be explained by the loss of p53 functions. However, the negative impact of high levels of ITGA1 may be counteracted by constitutive activation of EGFR signaling by mutation. This interpretation of the findings was already described in the first version of the manuscript.

**Comment 5: Section around line 236: What is the nature of the mutation that is introduced into the transduced p53 and how does this address mechanism? Is this mutant still able to bind DNA and transactivate? This section is confusing and needs to be clarified.**

Our answer: The transduced mutated p53 gene encode wild-type protein. Indeed, the DNA mutations were introduced at third nucleotide of a few codons, without changing the amino acids. These mutations in DNA in exogenous p53 gene, is not targeted by the guide RNA that was designed on the endogenous p53 gene sequence. We modified the text to better explain the followed strategy (page 11, line 249 and page 12, lines 250-51).

**Comment 6: Figure 3B: CRISPR-p53 only reduces p53 levels by 50% which is not very efficient. Why is it so inefficient?**

Our answer: The efficiency of CRISPR-p53 plasmids in decreasing p53 levels may be explained by the fact that we performed transient transfection experiments. However, even in these conditions we observed a significant increase of ITGA1 expression.

Reviewer #2

Major comments

**Comment 1: Figure 2 and onwards. All assays throughout are performed in HPV 38 positive cells. Interaction assays and CHIP assays should also be done for comparison in HPV negative cells to show clear stimulation by HPV38 E6/E7.**

Our answer: As requested, in the new version of the manuscript we have added p53 ChIP assay in ITGA1 promoter in primary human keratinocytes. p53 is not recruited to ITGA1 promoter in these cells (please see new Fig. 2C and additional text, page 7, lines 144-45).

**Comment 2: Figure 2C. The DNMT signal is marginal. What happens if cells are transfected with siRNA p53 or siRNA DNMT. This is alluded to later but it would be good to know which protein is recruiting which?**

Our answer: Our data findings from ChIP experiments showed that both proteins are required for their recruitment to ITGA1 promoter. In fact, inhibition of p53 by pifithrin, or silencing DNMT1 by siRNA, strongly inhibits the recruitment of both cellular proteins (please see Fig. 2F and I, in the new version of the manuscript).

**Comment 3: What happens with the band shift assays if phospho-mimic S392 p53 is included?**

Our answer: We agree with the reviewers that additional experiments with A392 or E392 p53 mutants will be very valuable. Unfortunately, due the confinement and restrictions in France, our laboratories are not fully operative and these experiments will require at least 2-3 additional months. Therefore, we would like to propose to conduct these experiments in future studies.

**Comment 4: Fig.S1B. This is critical data as all previous work has focussed on the role of DeltaNp73 – relevant siRNA approaches should be shown.**

Our answer: The majority of ΔNp73α sequence is shared with many p73 isoforms, except a small region at the N-terminus that is only present in ΔNp73α. The anti-sense ΔNp73α oligonucleotide used for down-regulating ΔNp73α expression targets the N-terminal regions. Unfortunately, we tried several siRNAs that target this region, but we did not manage to efficiently decrease ΔNp73α expression. In contrast, anti-sense RNA showed to be efficient and reproducible methods to decrease ΔNp73α protein levels, as shown in Figure S1B.

To further evaluate the role of ΔNp73α in ITGA1 expression, we performed ChIP experiments and showed that ΔNp73α is not recruited to IGA1 promoter in 38HK (please see new figure S1C and new text, page 9, lines 182-83).

**Comment 5: Figure 3. This should have HPV negative cells for comparison.**

Our answer: As requested by the reviewer in the comment 2, we provide evidence that p53 is not recruited to ITGA1 promoter in primary human keratinocytes (please see new Fig. 2C). Based on these findings, further analyses on the role of p53 in the regulation of ITGA1 expression in these cells may not provide additional insights.

**Comment 6: Figure 4. What happens if mutant S392A p53 is included in these assays – and/or the phospho mimic.**

**As the data stands it shows that phosphorylated S392 is bound but it does not show that it is phosphorylation at that specific site which is responsible. Is activation of PKR due to E6 or E7 or both? Assays using cells expressing them singly would be useful.**

Our answer: As state in our answer to the comment 3, we did not perform any experiments with p53 S392 mutants.

Regarding the second question, we have added a new panel in Figure 4 (C) that shows the impact of HPV38 E6 or E7 on PKR activation. Both viral proteins are able to activate PKR in U2OS cells. We observed that E7 alone is much more efficient than E6 or E6/E7 expressed as polycistronic RNA. It is possible that this phenomenon is due to a more efficient translation of E7 in comparison to the E6/E7 mRNA. A new text has been added (page 9, lines 194-98).

**Comment 7: Figure 5A. The co.ip data is weak. Again mutants of p53 at S392 would help clarify the molecular details.**

Our answer: please see our answer to comment 3.

**Comment 8: Throughout there appears to be two PKR bands on the blots – which is the bona fide protein. A simple siRNA should be performed to clarify which band is PKR.**

Our answer: Several isoform of PKR have been described in previous studies (e.g. Barber et al. Mol Cell Biol. 1995 15:3138-46; Blalock et al. J Cell Physiol. 2014 229:1047-60; Li et al.  J Biol Chem. 2001 276:13881-90; Blalock et al. 2011 25:236-45). For the immunoblottings we have used a PKR monoclonal antibodies that recognizes protein bands of the expected molecular weights: (i) Recombinant Anti-PKR (phospho T446) antibody [E120] (ab32036) from Abcam: <https://www.abcam.com/pkr-phospho-t446-antibody-e120-ab32036.html>; (ii) PKR (D7F7) Rabbit mAb #12297 from Cell Signaling: https://www.cellsignal.com/products/primary-antibodies/pkr-d7f7-rabbit-mab/12297).

**Comment 9: Figure 5B. I don’t understand why the pS392 form of p53 co.ips better than total protein – I would have thought the phospho form would no longer be bound. Again mutants of p53 would help clarify what is occurring here.**

Our answer: In our opinion, the signal reflects a better affinity of the anti S392 p53 antibody used in the immunoblotting, in comparison to the antibody against the total p53 form. Our data also indicate that the PKR/p53 interaction is not influence by the phosphorylation of S392 (please see Fig. 5B, similar amount of total p53 form was found associated in cells culture with or without PKR inhibitor).

**Comment 10: Figure 5D. This is critical to the whole story – a phosphomimic/phospho destroyed is really essential to showing that it is phosphorylation at that site which is critical**.

Our answer: Please our response to comment 3.

Reviewer #3

Minor comments

**Comment 1: Sentence in lines 65-67 does not make sense and needs editing.
Line 101 - sentence needs editing.**

Our answer: As requested, both sentences have been modified.

**Comment 2: The downregulation of ITGA1 protein by E6/E7 expression is demonstrated by immunofluorescence. This is acceptable although I wonder why they did not show this by Western blotting. Nonetheless, these IF data would be strengthened by quantification of multiple images which can be easily achieved using ImageJ.**

Our answer: In the new version of the manuscript, we have added ITGA1 immunoblottings that show the decrease of ITGA1 protein levels in HK or hTERT-HK expressing HPV38 E6/E7 (new Figs 1C and 1E), confirming the immunofluorescence staining.

**Comment 3: The binding of DNMT1 to the p53-RE2 region of the ITGA1 promoter shown by oligo pull-down in figure 2C is not very convincing. The conclusion would be strengthened by quantification of multiple pull down experiments to show consistency.
Why does CRISPR/Cas9 knockout of p53 only result in a small decrease in p53 mRNA abundance (Fig 3B). This reduction does reach significance but I would have expected a more dramatic decrease.**

Our answer: As requested, the quantification of three independent DNA-pull down assays has been added and confirmed the original findings (please see new Fig. 2D).

**Comment 4: How specific is the T446-PKR antibody used in the ChIP-reChIP assay shown in figure 5C and WB shown in figure 5D? Please provide a reference for a study demonstrating specificity.**

Our answer: To perform the ChIP-reChIP assay we used a rabbit monoclonal PKR antibody that only detects PKR phosphorylation on Threonine 446. This antibody is certified for ChIP assay as reported in its datasheet (Recombinant Anti-PKR (phospho T446) antibody [E120] (ab32036) from Abcam: <https://www.abcam.com/pkr-phospho-t446-antibody-e120-ab32036.html>).

**Comment 5: The authors state that the sucrose fractionation shown in Figure 5D demonstrates a trimeric complex containing p53/PKR/DNMT1. While the association between PKR and p53 is suggested by this experiment, the association of DNMT1 is less clear. Indeed, the treatment of cells with PKR inhibitor 2AP does not obviously alter DNMT1 fractionation. The interaction between p53 and DNMT1 is not clearly shown in the present study and I do not see sufficient evidence to conclude that this interaction is regulated by PKR phosphorylation of p53 on S392 as stated in lines 211-212.**

**The flow cytometry data shown in figure 6B are not clear – the histograms are too pale and hard to see.**

Our answer: To corroborate our initial findings we have added a p53 immunoprecipitation that shows the p53/DNMT1 interaction (Fig. 2H). Thus, the association between these two proteins is supported in our manuscript by (i) immunoprecipitation (Fig. 2H); (ii) ChIP/Re-ChIP (Fig. 2G) and (iii) and sucrose gradient fractionation followed by p53 immunoprecipitation. Regarding the last experiment, fractions 7-9 show the DNMT1 band that disappears upon inhibition of PKR by 2AP. Of note, the p53/DNMT1 interaction is in agreement with independent studies, as discussed in manuscript (please see Esteve et al. Proc Natl Acad Sci U S A. 2005;102(4):1000-5).

As requested, Fig. 6B (flow cytometry profile) has been improved.

**Comment 6: The Western blot shown in Figure 7E should be improved – at present there is no clear separation between the bands and it is therefore difficult to see the sequential reduction in HA-p53 expression following TMX treatment.**

Our answer: To provide a better imagine of the same experiment, we have substituted the panel of Fig. 7E by a p53 immunoblotting with a lower exposure.

We would like to thank you and the reviewers again for the valuable comments that considerably improved our original manuscript.

Yours sincerely,

Massimo Tommasino
